# Combination of Specific Vascular Lasers and Vascular Intense Pulsed Light Improves Facial Telangiectasias and Redness

**DOI:** 10.3390/medicina58050651

**Published:** 2022-05-11

**Authors:** Luigi Bennardo, Cataldo Patruno, Elena Zappia, Federica Tamburi, Mario Sannino, Francesca Negosanti, Steven Paul Nisticò, Giovanni Cannarozzo

**Affiliations:** 1 Department of Health Sciences, Magna Graecia University, 88100 Catanzaro, Italy; luigibennardo10@gmail.com (L.B.); cataldopatruno@libero.it (C.P.); elena.zappia@hotmail.it (E.Z.); federica.tamburi@gmail.com (F.T.); steven.nistico@gmail.com (S.P.N.); 2 Department of Systems Medicine, Tor Vergata University, 00100 Rome, Italy; dr.mariosannino@gmail.com; 3 Dermatologic Center “Villa Bella-Antiaging Care Group”, 40126 Bologna, Italy; francesca.negosanti@gmail.com

**Keywords:** vascular IPL, Nd:YAG laser, telangiectasias

## Abstract

*Background and objectives:* Facial telangiectasias are dilated blood vessels that can represent a cosmetic issue for patients. They may be associated with other conditions, such as rosacea. Laser and light treatments are nowadays becoming a cornerstone in the management of these lesions. *Materials and Methods:* In total, 68 patients seeking medical treatment for facial telangiectasias were enrolled from 1 March 2019 to 1 March 2020 at the Dermatological Unit of Magna Graecia University (Catanzaro, Italy). A protocol consisting of a 1064 Nd:YAG laser for darker blue telangiectasias and 532 nm Nd:YAG for red lesions followed by intense pulsed light with an optimized spectrum for vascular lesion 3 weeks after the first procedure was proposed. A three-month follow-up visit assessed patient’s satisfaction using a visual analog scale (VAS). Two dermatologists measured clinical results using a 4-point scale, comparing pictures before treatment and at follow-up. *Results:* A total of 68 patients (32 males and 36 females) completed the study, performing all requested treatments. No severe side effects were reported. Patient satisfaction was very high (8.15 ± 1.05 out of a 10-point VAS scale), as well as dermatologists’ clinical evaluations (2.19 ± 0.74 out of 3). *Conclusions:* The combination of vascular lasers and Vascular Intense Pulsed Light acting specifically on small blood vessels may help to improve the aesthetic outcome, reducing side effects. A prospective study with a larger number of participants will be necessary to confirm this study’s findings.

## 1. Introduction

Facial telangiectasias are small blood vessels, typically measuring 0.1–1.0 mm in diameter, which may be of venular, capillary, or arteriolar origin. They are a common cosmetic problem, with 92% of adults displaying mild or limited lesions, and people affected by this condition search for medical help more and more often [1]. Usually, they may be associated with other diseases, such as rosacea or poikiloderma of Civatte or to a variety of factors including genetic predisposition, gravity, pregnancy, and trauma [2,3]. They more commonly affect people aged 30–50 years old and with lighter skin phototypes, although everyone can be affected [4]. Various therapies have been proposed to manage this cosmetic problem, such as topical creams, laser and light devices, with variable results [5,6]. Nowadays, topical gels based on alpha-adrenergic agonists such as brimodine have been proposed to treat erythrosis and small facial telangiectasias. However, their effect is temporary, lasting at most 12 h, and they do not affect larger caliber telangiectasias [7,8]. To target larger vessels and have more lasting cosmetic results, laser and lights sources have been proposed. Specifically, the 1064 nanometer (nm) neodymium-doped yttrium aluminum garnet (Nd YAG) laser seems to be the best device in managing larger vessels, whether concerning the face or other body areas, while lasers targeting in the 500–600 nm spectrum, such as the 585–595 nm Pulsed Dye (PDL) and the 532 nm Nd YAG laser, have been proposed to treat erythrosis, smaller vessels, and telangiectasias. Intense Pulsed Light (IPL) has been proposed to manage erythrosis with satisfactory results [9,10]. The Nd:YAG laser is responsible for an adequate absorption at the blood vessel levels (where hemoglobin is the main chromophore) and for a deeper penetration through the skin in comparison to shorter wavelengths and so being able to penetrate dermal and subdermal vessels. Moreover, it benefits from a low absorption of melanin and therefore it is suitable for treating a large variety of phototypes with decreased risk of depigmentation at the treated site [11]. IPL delivers an intense, broad-spectrum pulse of light, generally in the spectral range of 400–1200 nm, although various filters may be used to reduce the wavelength spectrum and make the light more selective for a single target. It has been proposed in the management of various conditions, such as photoaging and rosacea [12,13,14].

In this paper, we will analyze the results following a novel combination of IPL, 532 nm, and 1064 nm Nd YAG lasers in managing facial erythrosis.

## 2. Materials and Methods

Patients presenting for the removal of facial telangiectasias and erythrosis to the Dermatology laser Unit of Magna Graecia University, Catanzaro, from 1 March 2019 to 1 March 2020 were retrospectively enrolled in this study. Patients suffering from hypersensitivity to visible light, taking medication capable of increasing light sensitivity, such as sulfonamides, sulfonylureas, phenothiazines, and contraceptives, having performed in the last three months any laser or light treatment, reporting the use of topical medications such as retinoids, salicylic/glycolic acid, antibiotics, being pregnant or breastfeeding, as well as reporting neoplasms or other systemic conditions were excluded from the study. All patients signed an informed consent on the risk of the procedure. The procedure consisted of a session of a 532 nm Nd Yag laser (Flash handpiece of Luxea, DEKA M.E.L.A., Calenzano, Italy) performed on telangiectasias appearing red at the multispectral analysis (Antera3D, Miravex ltd., Dublin, Ireland) with the following parameters: 8–10 J/cm^2^ at 6–15 ms pulse duration with a spot diameter of 6–10 mm. A session of a 1064 nm Nd Yag laser (Sparks handpiece of Luxea, DEKA M.E.L.A., Calenzano, Italy) was performed on telangiectasias appearing blue or darker at the multispectral analysis with the following parameters: 120–150 J/cm^2^, 4 mm spot size, and a 7–15 ms single pulse. Three weeks after this first procedure, a session of IPL with a 550 nm filter (Luxea, DEKA M.E.L.A., Calenzano, Italy) using a transparent gel with the following parameters was performed: fluence ranging between 14 and 18 J/cm^2^, a first pulse of 4 ms, a second impulse of 6 ms, and a delay of 10 ms between pulses. External air-flow cryogen cooling at 5 °C (SmartCryo 6, DEKA M.E.L.A., Calenzano, Italy) was present during all the procedures. No anesthetic ointment or subcutaneous injection was performed, as the pain was considered bearable by all patients. Patients had to apply an SPF 50 protection cream every morning up to clinical follow-up. Follow-up was performed three months after the IPL session. Pictures were taken before treatments and at three months follow-up using the same camera (Nikon 5600d, Nikon Corporation, Minato City, Tokyo, Japan) and the same shooting settings. Considering the absence of a specific scale to evaluate facial telangiectasias, two independent researchers (L.B. and G.C.) evaluated the pictures giving a score on a 4-point scale (with 0, no improvement/worsening of the condition; 1 slight improvement of the condition; 2 moderate improvements; and 3 excellent improvement/disappearance of the condition). A Visual Analogue Scale (VAS) from 1 to 10 was administered to the patients at the follow-up to measure patient satisfaction.

## 3. Results

**Figure 1:**
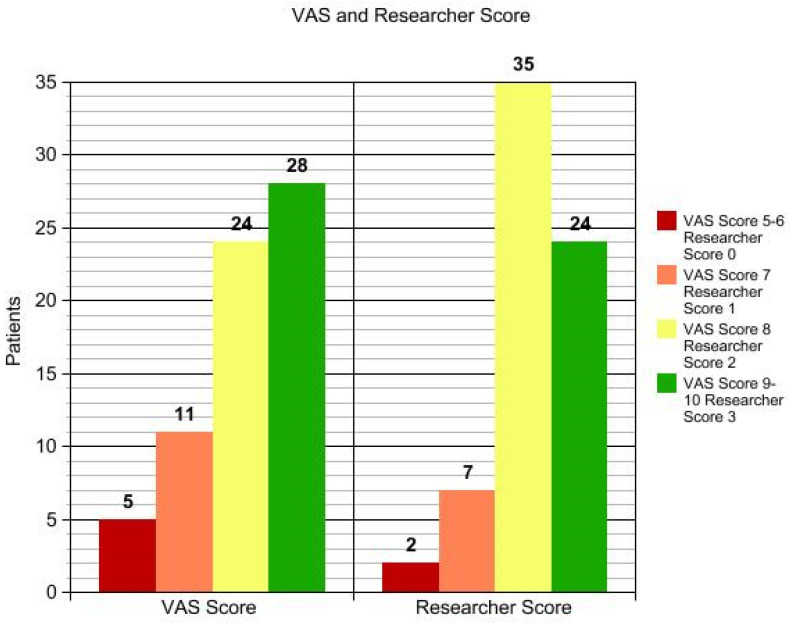
VAS and researcher score.

**Figure 2:**
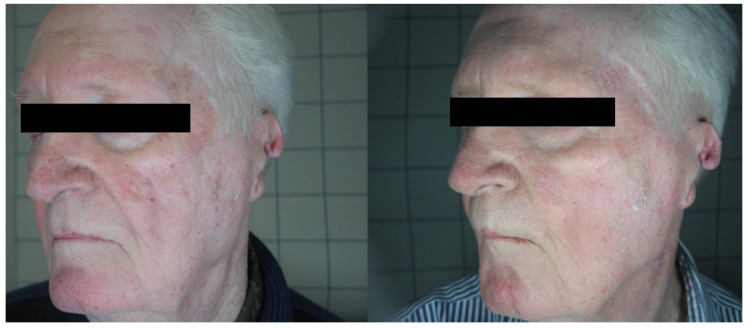
Patient n. 33 before (left) and 3 months after IPL treatment.

**Figure 3:**
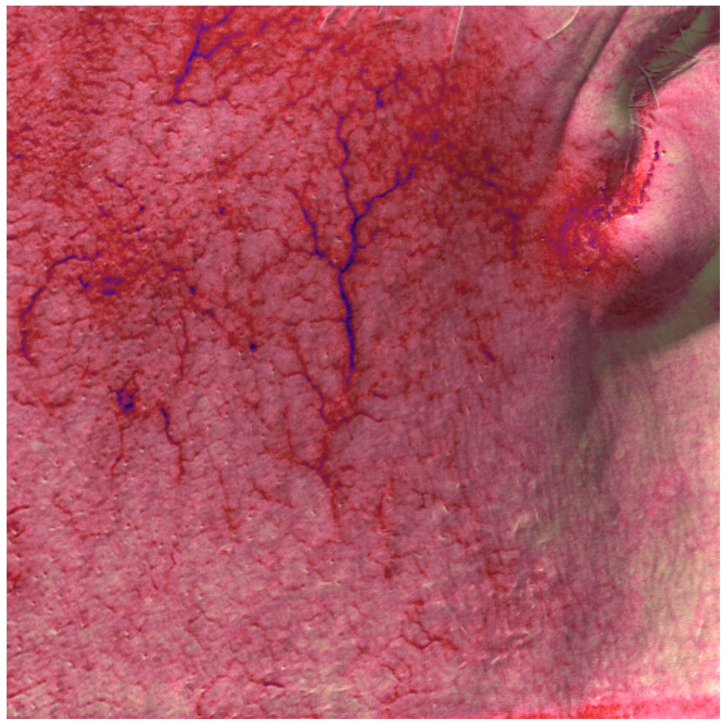
Patient n. 33 multispectral analysis before all treatments.

**Figure 4:**
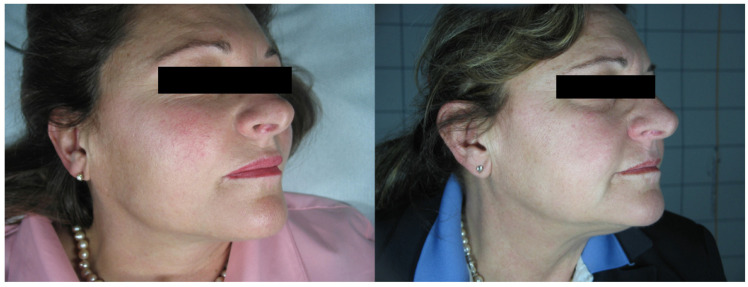
Patient n. 34 before (left) and 3 months after IPL treatment.

**Figure 5:**
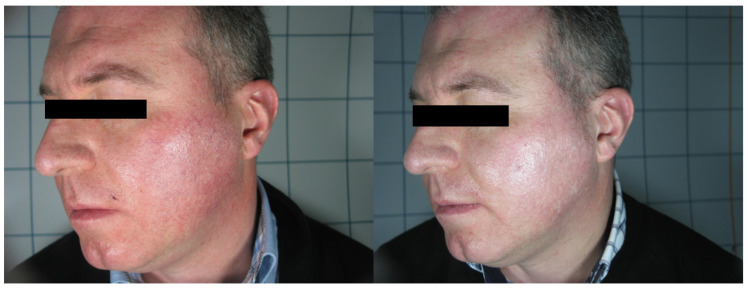
Patient n. 46 before (left) and 3 months after IPL treatment.

**Figure 6:**
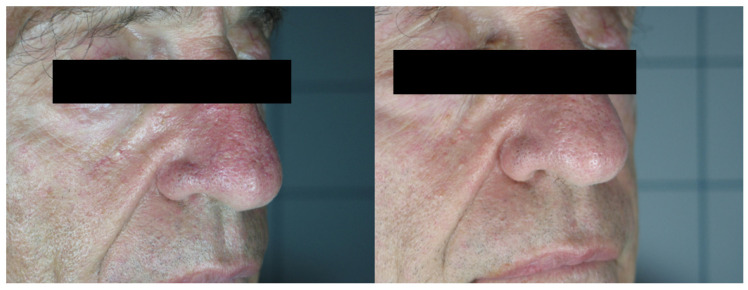
Patient n. 31 before (left) and 3 months after IPL treatment.

In total, 68 patients (mean age 48.34 ± 10.56), 36 females and 32 males, were enrolled in the study and underwent the procedure. All patients were classified according to Fitzpatrick’s phototype scale. Almost all patients reported no to minimal pain during the procedure, with just four patients reporting moderate pain. No anesthetic procedure was, however, necessary. No significant side effects were reported. In two cases, some crusts and scales appeared after the procedure, spontaneously resolving after two weeks with no post-inflammatory hyper/hypopigmentation. The mean satisfaction score reported by the patients at VAS was 8.15 ± 1.05, with just one patient reporting an unsatisfactory result. (Figure 1, Figure 2, Figure 3 and Figure 4). The dermatologist evaluation comparing the pictures showed a mean score of 2.19 ± 0.74, with an overall good response to treatment (Figure 1, Figure 2, Figure 3, Figure 4, Figure 5 and Figure 6). No statistically significant difference in outcome in terms of gender or age was detected. Patient characteristics are reported in Table 1.

## 4. Discussion

Telangiectasias are visible dilated blood vessels with diameters normally ranging from 0.1 to 1.5 mm (mm); they may appear spontaneously or be secondary to a medical condition (for example, rosacea) or predisposing factors (light exposure, steroid intake/topical application, alcohol intake, etc.) [15]. They may result from a superficialization of vessels of the deep dermis, mostly following local inflammation, with a reduction in the upper dermal connective tissue integrity [16].

The presence of these lesions, often associated with erythrosis on the face, may be a significant cause of stress and laser systems showing affinity to hemoglobin have become the mainstay in the treatment of telangiectatic lesions. They work thanks to a process of selective phototermolisis, where a substance absorbs light of a specific wavelength better than others. Oxyhemoglobin has a peak of absorbance at 577 nm, so vascular lasers such as 595 nm PDL and 532 nm Nd YAG emit very near to this peak, guaranteeing a good absorbance and a selective action on vessels [17,18].

Lower wavelength lights tend not to be absorbed selectively by hemoglobin and to hit skin very superficially, generating an anti-inflammatory effect that may be used in the management of various inflammatory conditions [19,20]. Lights and laser with longer wavelengths tend to go deeper in the skin and may be effectively used to target collagen and melanin [19,20,21,22,23].

Success using IPL to treat telangiectasias has been widely reported.

In the first years of the new millennia, different filters were proposed to improve the effectiveness of IPL for facial telangiectasias [24].

A Chinese study compared PDL and IPL with different filters in treating facial telangiectasias, showing no difference in results between the 595 nm laser and IPL with vascular filters (530–650 and 900–1200 nm) [25]. Other researchers have also observed similar results on facial and body telangiectasias [26,27].

Another study proposed using a narrow band (500–600 nm) IPL light in the treatment of facial telangiectasias with good results [28].

An Italian group compared the effectiveness of rhodamine IPL in managing facial telangiectasias compared to classical IPL, showing better effectiveness, although not reaching statistical significance [29].

Additionally, other studies report the use of vascular filters to enhance the effectiveness of IPL [30]. Single wavelength 540 nm IPL has also been proposed to treat telangiectasias and late-stage rosacea, showing efficacy in managing this condition [31].

Although traditionally used to treat vascular malformations [32] and in the management of scar formation [33,34], PDL has also been reported in the treatment of facial telangiectasias. A case series suggested that a multipass sub-purpuric approach may be as effective as the traditional single-pass purpuric approach, reducing the laser-associated side effects [35].

Longer wavelength lasers, such as the 1064 Nd YAG laser, may be used to treat deeper lesions, such as darker or larger diameter telangiectasias, due to their ability to penetrate the dermis and not be absorbed by superficial skin layers [36,37]. Due to its skin penetration, the Nd:YAG laser may also be used with nanosecond or picosecond pulses to manage tattoos and hyperpigmentation [38].

The use of Nd YAG laser in facial telangiectasias has been widely reported. A diode-pumped frequency-doubled 532 nm Nd:YAG laser has been proposed to treat facial telangiectasias in 66 patients, achieving a 75/100% clearance of the lesions in 62 patients, with a low profile of undesired effects that can be well tolerated by patients [39].

A German group treated 17 patients with facial telangiectasias affected by hereditary hemorrhagic telangiectasia with outstanding cosmetic results [40].

A case series reported the use of a 1064 nm Nd:YAG laser in managing facial telangiectasias with a diameter variable from 0.3 to 2 mm. Three pulse widths (3, 20, and 60 ms) were reported, with fluences varying depending on vessel size and response. Mean vessel clearance was reported by three unbiased dermatologists and was assessed at 26–50% in half of patients and 50–75% of the lesions in the other half [41].

A Finnish group performed a split-face double-blinded study that compared the treatment efficacy of a 532 nm KTP laser with a new 585 nm laser in the management of cheek telangiectasias, showing similar results with both lasers, although physicians evaluated the KTP-treated side as the most improved. Pain score reported by patients was also higher on the 585 nm laser side [42]. Similar results were also obtained comparing the KTP laser with the 595 nm laser, showing that the 532 nm laser was slightly more effective but reporting a relatively higher rate of side effects [43].

Twenty subjects with light Fitzpatrick phototypes were treated with a micropulse (0.65 ms) 1064 nm Nd:YAG laser in two different sessions, showing a total clearance in two patients, a significant reduction in vessels in 15 patients, and a lighter effect only in three subjects, without reporting any side effects [44].

A Chinese group tried to sequentially use the Nd:YAG laser after IPL in the management of facial telangiectasias, comparing them with single treatment results performed in two sessions separated by three days [45]. In the split-face study, half of the face was treated in the same session with both therapies. The other side was treated initially with IPL and then, after three days, with the Nd:YAG laser. The 24 participants were then evaluated at four weeks of follow-up. Outcome measures were clinical efficacy, using a 7-point Telangiectasia Grading Score, pain, adverse effects, patient satisfaction, and preferred treatment. Although patients were satisfied with both treatments, clinical efficacy was significantly superior in combined therapy, as well as side effects such as erythema, purpura, and edema were significantly superior [45].

This paper aims to report the sequential use of the Nd YAG laser with IPL to treat telangiectasias. The first treatment with the 532 nm/1064 nm Nd YAG laser according to the telangiectasia’s multispectral appearance aims to lead to the reduction of larger lesions, while the sequential second disactivated IPL treatment has the goal to reduce the redness and all the tiny and superficial vessels not addressed by laser treatment causing erythrosis. To our knowledge, this is the first time that the Nd:YAG laser and rhodamine IPL have been proposed in such a sequential treatment in the management of facial telangiectasias, showing considerable efficacy. Although further comparison studies will be necessary we think that this combination technique will guarantee superior results compared to any other non-combined procedure, with results slightly better than other Nd:Yag and IPL combination techniques, possibly becoming a mainstay (when available) in the management of facial telangiectasias and redness.

## 5. Conclusions

Facial telangiectasias are a cosmetic issue impacting the psychological health of affected patients. Various laser treatments have been proposed, and very few combination treatments have currently been proposed in the medical literature. The sequential use of a 532/1064 nm Nd:YAG laser and IPL has proven to be very effective in the management of facial telangiectasias and erythrosis; of course, both procedures are very operator-dependent, and an expert researcher familiar with these devices should perform the treatment, in order to reduce the rate of side effects and to improve efficacy. The main limitation of this study includes the relatively low number of patients and the absence of a control group performing monotherapy. A prospective trial with a control group would be necessary to confirm the superiority of this new combined technique compared to other available treatments.

## Figures and Tables

**Table 1:** Patient characteristics.

Patient Number	Age	Sex	Fitzpatrick Phototype	VAS Score	Researcher Score	Patient Number	Age	Sex	Fitzpatrick Phototype	VAS Score	Researcher Score
1	61	f	2	8	2	35	49	m	1	8	2
2	43	m	3	6	1	36	56	f	2	9	3
3	48	f	2	9	3	37	37	m	3	8	2
4	54	m	2	9	3	38	52	f	3	9	2
5	58	f	3	9	2	39	58	f	4	9	3
6	51	m	3	7	2	40	42	m	3	8	2
7	34	m	2	8	2	41	51	m	2	7	2
8	27	f	1	10	3	42	45	f	3	8	2
9	39	m	2	7	2	43	35	m	3	8	2
10	58	f	3	8	3	44	38	m	2	9	3
11	52	m	4	9	2	45	39	f	4	8	2
12	63	f	3	9	3	46	46	m	3	8	2
13	42	m	4	6	1	47	73	f	4	9	3
14	49	f	4	7	2	48	24	f	3	9	2
15	58	m	2	8	2	49	38	m	1	10	3
16	43	f	1	9	3	50	46	m	4	6	1
17	38	m	2	8	2	51	49	f	5	6	0
18	36	f	1	9	3	52	43	m	3	7	2
19	62	m	3	8	2	53	38	f	4	8	1
20	56	f	2	9	3	54	53	f	3	9	2
21	58	m	3	9	3	55	58	m	3	8	2
22	43	m	3	8	2	56	39	f	4	7	2
23	46	f	3	7	2	57	48	m	3	9	3
24	34	f	4	7	1	58	53	f	3	8	2
25	49	f	3	8	2	59	36	m	3	7	1
26	52	m	4	9	3	60	38	f	3	8	2
27	67	m	3	8	2	61	48	m	2	9	3
28	52	f	4	10	3	62	43	f	4	5	0
29	25	f	4	9	2	63	52	m	2	10	3
30	63	f	3	9	3	64	58	f	3	9	3
31	64	m	3	8	3	65	57	f	2	8	2
32	36	f	4	7	2	66	54	f	3	7	1
33	71	m	2	8	2	67	52	m	3	8	2
34	48	f	3	9	3	68	59	f	3	9	3

## Data Availability

Data are available from the corresponding author upon reasonable request.
